# Experiences of using an activating spinal orthosis in women with osteoporosis and back pain in primary care

**DOI:** 10.1007/s11657-020-00754-z

**Published:** 2020-10-29

**Authors:** Christina Kaijser Alin, Nathalie Frisendahl, Ann-Charlotte Grahn Kronhed, Helena Salminen

**Affiliations:** 1 Department of Neurobiology, Care Sciences and Society, Division of Family Medicine and Primary Care, Karolinska Institutet, Solna, Sweden; 2 Department of Neurobiology, Care Sciences and Society, Division of Physiotherapy, Solna, Sweden; 3 Rehab Väst, Local Health Care Services in the West of Östergötland, Mjölby, Sweden; 4 Division of Physiotherapy, Department of Medical and Health Sciences, Linköping University, Linköping, Sweden; 5 Academic Primary Health Care Centre Stockholm, Stockholm, Sweden

**Keywords:** Osteoporosis, Spinal orthosis, Older women, Qualitative study, Back pain, Back extensor strength

## Abstract

***Summary*:**

Women with osteoporosis and back pain took part in focus group interviews and described their experiences of using and handling an activating spinal orthosis. The women described the back orthosis as being like a “close friend”, a support in everyday life and a reminder to maintain a good posture.

**Purpose:**

The purpose of this study was to describe and gain a deeper understanding of the views of older women with osteoporosis and back pain seeking primary care regarding their use and handling of an activating spinal orthosis.

**Method:**

We chose a qualitative method whereby information was gathered via focus group interviews and analysed using inductive content analysis. Women who previously participated in a randomised controlled trial and wore an activating spinal orthosis for 6 months were asked. Out of 31 women, 18 agreed to participate. Five focus group interviews were conducted.

**Results:**

The analysis resulted in an overall theme in which the experiences of wearing the spinal orthosis were described as follows: “A well-adapted spinal orthosis could develop into a long-lasting friendship that provided support and help in daily life”. The overall theme was based on three main categories: *impact on daily life*, *individual adaptation* and *personal relationship*. The main categories were well differentiated from each other but had an interdependency. All three categories involved cases in which the spinal orthosis was perceived as relieving symptoms and making daily life easier, as well as when it was perceived as being hard to manage and provided no symptom relief.

**Conclusion:**

In older women with osteoporosis and back pain, an activating spinal orthosis could be perceived as being a “close friend” and a support in everyday life. To facilitate acceptance of the spinal orthosis, it was important for it to be well adapted and for follow-ups to be carried out regularly.

**Electronic supplementary material:**

The online version of this article (10.1007/s11657-020-00754-z) contains supplementary material, which is available to authorized users.

## Introduction

Osteoporosis and back pain are common health problems among older women and common causes for visits in primary care. In addition to the medicinal relief of back pain, it has been shown that training the back extensors can lead to pain relief, improved posture and lung function, reduced fall risk and reduced risk of vertebral fractures [1, 2]. Training the back extensor muscles also gives increased confidence in the ability to perform daily activities, as well as improved health-related quality of life [3]. For patients with osteoporosis and back pain, back extensor training can be carried out in various ways. Exercise equipment at a gym, rubber bands and specific back training (“back-ups”) can all have a good effect, including increased back muscle strength, reduced back pain and improved health-related quality of life, while also making it easier to perform daily activities [4, 5]. Reduced strength in the back extensors can also put a person at risk of future vertebral fractures [6, 7].

Individuals with osteoporosis and vertebral fractures have described a life characterised by periods of back pain that is limiting and which reduces their confidence in their ability to perform daily activities. Moreover, individuals are constantly concerned that their back pain will return, which creates a sense of uncertainty about the future. Women may also feel that healthcare professionals do not have a sufficient understanding of their problems and feelings and that their problems are not taken seriously [8, 9].

A spinal orthosis has been developed that can activate the back muscles while also providing support for the back [10]. This spinal orthosis has been used in patients with osteoporosis and back pain, as well as in the subacute phase of rehabilitation after a vertebral fracture. It has increased the strength of the back extensor muscles, reduced back pain and even improved posture. Part of the increase in back extensor strength can possibly be attributed to spontaneous posttraumatic healing, but it has also been shown that the orthosis has relieved pain and improved posture [10–16].

The orthosis is designed like a backpack, with straps around the shoulders and button over the pelvis/lower part of the abdomen. By means of a feedback system, the aim is to activate into an upright position and stabilise the lumbar region by means of pressure across the lower part of the abdomen. A metal rod on the back is inserted into the orthosis, which is adjusted to the person’s spine in an upright position as possible. This adaptation was carried out by an orthopaedic technician. When the person is wearing the spinal orthosis, the back is allowed some mobility. Upon flexion of the back, the feedback system causes the shoulder straps to activate the back extensor muscles continuously in order to straighten the back.

In a previous study, we showed that a spinal orthosis can be used as an alternative training method, which increases the possibility of customising the training to suit each patient [13]. However, a deeper understanding of an activating spinal orthosis is required, as well as feedback from patients regarding their use of this device. At present, there have been no qualitative studies on people’s experience of a spinal orthosis.

The purpose of this study was to gain a deeper understanding of the experiences of women with osteoporosis and back pain with and without vertebral fractures in primary care regarding their use and handling of an activating spinal orthosis.

## Method

### Design

A qualitative inductive approach was applied to this study. We wanted a rich picture and understanding of the interviewees’ different perceptions of wearing an activating spinal orthosis called Spinomed (Medi AB, manufacturer). We chose focus group interviews as the method of data collection and qualitative content analysis as the method of data analysis [17, 18].

The focus group interviews proved to be both a useful and a suitable method since all interviewees shared common experiences of some kind and probably felt empowered and inspired by each other during the discussion [19, 20].

### Participants

Strategic selection was applied to this study. A group of older women with osteoporosis and back pain, with or without vertebral fractures, was selected. All vertebral fractures were of older dates. They had previously taken part in a randomised controlled trial (RCT) with three arms [13]. Background information was collected in the RCT, Table 1. The participants who were randomised to the spinal orthosis group had the orthosis fitted and individually adjusted by an orthopaedic technician. This was followed up by further adjustments after around 4 weeks. The women were informed that they should wear the orthosis at least 2 h per day for 6 months, which they did. All those women who completed the 6-month intervention period (*n* = 31) were asked whether they wished to participate in a focus group interview. They received a letter with a detailed description of how the focus group interview would be conducted. A number of women were also contacted by phone. A total of 18 women agreed to participate in the focus group interview and 13 women declined to participate due to holidays, illness or conflicts with activities or work.

**Table 1:** Characteristics of the participants (*n* = 18)

		Baseline		After 12 months	
		Mean	(SD)	Mean	(SD)
Height present	(cm)	162	(6.3)	161	(6.7)
Height in youth	(cm)	166	(5.3)		
FVC	(litre)	2.9	(0.7)	2.9	(0.7)
Back muscle extensor strength	(Newton)	71.4	(39.0)	80.4	(45.6)
		Median	(IQR)	Median	(IQR)
Age	(years)	77	(67–81)	78	(68–82)
Back pain, last week Visual analogue scale (VAS)	(mm)	52.5	(31–76)	40	(20–68)
Back pain, last week Borg CR-10	(0–10)	4	(3–5)	3	(2–6)
		*n*	%	*n*	%
Vertebral fracture X-ray		10	55		
Kyphotic index ≥ 13		10	55	11	61
Bone-specific drugs		10	55	11	61
Smoking		None		None	
Time spent outdoors ≥ 30 min/day		15	78	15	78
Living alone		11	55	11	55
Living in their own homes		18	100	18	100

*FVC* forced vital capacity

### Data collection

The data were collected through five focus group interviews conducted from November 2014 to January 2015. The number of interviewees at the various interview sessions varied from two to five. The interviews took place at a unit at the Sabbatsberg Hospital in Stockholm. All interviewees had previously visited the unit, so the location felt familiar and safe. All but one of the interviews were conducted by two co-authors (CKA, NF). One served as moderator and the other as an observer. The first focus group had a third co-author (HS) as an observer and CKA as a moderator. The observer took notes about what happened during the interviews and also about what could not be captured on the recordings, for example the interaction between the respondents. A semi-structured interview guide was used as support during the interviews (see Appendix 1 in the Supplementary Material). The interview guide contained several open questions regarding thoughts about how the spinal orthosis affected the interviewees’ daily life and how their bodies felt, as well as thoughts about handling the orthosis. In order to create an interview atmosphere that fostered a sense of trust, the moderator and observer introduced themselves before the interview started, with a presentation of their professional background and their role in the study. The opening question was “Please describe your thoughts about the orthosis?” Follow-up questions were then asked in order to delve deeper into the answers until the questions were exhausted. The time required for the interviews varied between 29 and 55 min, depending on the number of participants.

### Data analysis

There was a discussion between the moderator and the observer as a first impression of what was perceived during the interview before the first step with transcription in the analysis process. The recordings of the five focus group interviews were transcribed verbatim by one of the co-authors (NF). Inductive qualitative content analysis was used as the method of text analysis [17, 18]. This method was chosen in order to achieve as rich and detailed picture as possible of the interviewees’ experience. Initially, all the text was read by the co-authors to enable them to familiarise themselves with the material. Analysis of the text then followed the process that applies to inductive qualitative content analysis [17]. To obtain objective results and investigate whether our analysis and perception of the text differed, the author and two co-authors began with an analysis of the interviews. Meaning-bearing units with similar content were extracted from the text and classified, condensed and coded to form subcategories and overarching categories. Analysis then began of all five interviews that the authors had coded and sorted into subcategories. Further analysis was performed jointly by all four authors, who reviewed the subcategories and developed these into three overarching categories. Finally, an overall theme covering all three categories was developed. An example of the analysis process can be found in the Appendix 2 in the Supplementary Material.

### Credibility

Credibility is a key concept in qualitative research based on five concepts: validity, reliability, subjectivity, transferability and authenticity [18, 21, 22]. To achieve validity in this study, the selection, design and method were described in detail. Reliability was increased through a large proportion of the work being performed jointly by two or more co-authors. To reduce subjectivity, coding and categorisation were performed by two authors and the analysis was then performed jointly by all co-authors. Two co-authors each authored a results section. These were then compared and merged into a final version by the entire research team.

### Ethical considerations

Ethical approval was obtained from the Ethical Review Board of Stockholm (Reg. no. 2011/142-31/3). All women who agreed to participate in the focus group interview received both oral and written information about the purpose of the study and they all signed an informed consent form. They were informed that their participation was voluntary and that they could withdraw at any time without this affecting their health care going forward.

## Results

The median age of the 18 women who participated in the focus group interviews was 78 years. Back pain over the last week was rated according to both Borg CR-10 and VAS [23, 24]. At the inclusion to the RCT-study, the back pain had to be graded to at least 30 mm on a VAS scale and to 2.5-3 according to the Borg CR-10 (moderate back pain) and participants had to have osteoporosis verified with a DXA measurement. This data was collected in the RCT [13]. See further Table 1.

### Overall theme

#### A well-adapted spinal orthosis could develop into a long-lasting friendship that provided support and help in daily life

The spinal orthosis proved to be capable of significantly impacting the individual’s everyday life by making it easier to perform daily activities. In order to have a positive effect, it needed to be adaptable and designed based on the individual’s needs.

During the interviews, it was found that the spinal orthosis could positively affect daily life insofar as it made the back feel better and less painful and it provided support and improved posture. Because of the positive effects, several interviewees wanted to continue using the spinal orthosis even after the trial had ended; they likened the relationship to a friendship. The prerequisites for a good relationship with the spinal orthosis related to the fitting and individual adjustment to the person’s back. In cases in which the spinal orthosis did not provide improved posture or support, the relationship was adversely affected.

The overall theme was based on three main categories: *impact on daily life*, *individual adaptation* and *personal relationship*. These categories, along with the associated subcategories, are shown in Table 2.

**Table 2:** Overall theme and the three main categories with their subcategories

Theme: “A well-adapted spinal orthosis could develop into a long-lasting friendship that provided support and help in daily life”	
Main category	Subcategory
Impact on daily life	Better posture Support Stronger back Pain relief Training equipment More reliable body control
Individual adaptation	Fitting Self-adjustment
Personal relationship	Expectations Notions of the orthosis Relationship over time Thoughts of the individual and reactions from those around them Thoughts about continuing the use of the orthosis

#### Impact on daily life

The interviewees indicated that the spinal orthosis had improved their posture and reminded them to slide their shoulders back in order to straighten their backs. It could also increase their awareness of their back and posture when performing various daily activities, even when the orthosis had been removed. One interviewee stated the following:

...but what I think I got out of it is that posture is an added bonus. I mean, when you wear the vest you get to know what it should feel like. You can also remember that particular posture even when the vest is off. (P3, interview 2, p. 2).

The interviewees indicated that their breathing was positively affected when their backs felt straighter. It felt easier to inhale. In order for the spinal orthosis to provide these positive experiences, some of the interviewees stated that the orthosis must be used regularly. Otherwise, the effect diminished quite quickly and the posture returned to its previous status.

The spinal orthosis could feel like a support to lean against or like a supporting hand. It could make it easier to travel on public transport because it made the back feel more stable. The support they felt enabled them also to keep going when they took long walks, as well as perform activities that put more strain on their backs. Day-to-day activities involving standing and walking, such as household chores, were easier.

Regular use of the spinal orthosis decreased symptoms such as a sense of fatigue and made it easier to keep their backs straighter. They could perform strenuous activities for longer periods and that they felt stronger. It felt like the muscles were working properly when they wore the spinal orthosis. One interviewee said:

I’m happy that I’ve grown so much stronger and can now do so much more than I’ve been able to over the last 20 years. I’ve been able to work for much longer periods both inside my home and out in my garden. And I’m now able to swim 500 metres compared to 100 metres before and I am also able to carry more. So, I think it has had a very positive effect on my strength and posture (P3, interview 5, p 2).

Back pain had been alleviated or toned down when the spinal orthosis was used and there had been a positive impact on daily life. Activities that were previously difficult could now be done with less or even no pain. For some interviewees, their back pain decreased quickly after the spinal orthosis was fitted, while others indicated that they had to wear it for some time before their back pain was alleviated. Some stated that they slept more peacefully at night because their backs had improved, while another stated that the orthosis had had no effect on their back pain.

Yes, I must agree with the others that it’s been fantastic because I’m always on the go. For example, I love to bake and if I don’t wear it the muscles below my ribs hurt so much. In fact, they hurt so much that I have to lie down. It’s the same when I’m hoovering (...). (P4, interview 1, p. 2)

A spinal orthosis could be a good aid to start training the back and help increase back strength. Combining the use of the spinal orthosis with other forms of training could also worked well. It promoted to good exercise habits.

(...) I’ve never managed strength training before and I think it’s simply the corset that helped me get started. (...) So my endurance and strength have improved. (P3, interview 5, p. 5)

The spinal orthosis could give a sense of security when performing various activities by preventing and counteracting the kind of movements in the back that should be avoided.

When I’m wearing the corset I feel more secure because it somehow prevents me from making unusual movements with my back. So using it is like a kind of insurance policy (P2, interview 5, p. 5)

#### Individual adaptation

Several interviewees stated how important it was for the spinal orthosis to be fitted individually and carefully so that it followed the curvature of the spine and felt comfortable and easy to wear. They also stated that it felt good when it fitted properly over the pelvis and the lumbar support was positioned correctly. It was also important to get detailed information about how the spinal orthosis should be fitted and handled and not to hurry the fitting process. Good communication about the fitting of the spinal orthosis was also important. Some interviewees stated that it was difficult to get a good fit to the back. The fitting process was made more difficult if their backs were severely deformed or kyphotic. One adjustment of the spinal orthosis was necessary after a period of use as their backs had become straighter or had changed in some other way. Some interviewees stated that the spinal orthosis felt comfortable from the start and that they liked it, while others thought it felt inflexible and thick.

I found it quite uncomfortable to wear in hot weather. The synthetic material makes you all sweaty. (P1, interview 1, p. 8)

When using the spinal orthosis, they had to adjust it regularly in order for it to fit well. It tended to ride up when they moved around, e.g. when hoovering, and even more so when doing work above shoulder height. The spinal orthosis then had to be opened, pulled down and buttoned again. Most interviewees agreed that some strength in the hands was needed to be able to button it so that it sat properly. Using the toilet could be perceived as cumbersome since the spinal orthosis had to be unbuttoned and removed.

I used it a lot when I was taking a stroll, etc. I had to move my body in many different directions and keep pulling the spinal orthosis down all the time (P5, interview 2, p. 4).

Several interviewees felt that the spinal orthosis had a strange shape and affected their appearance and made them look like they had a hunchback. For this reason, they chose to hide it, using a sweater or jacket, or to not use it at all when leaving the house because of the reactions and questions from those around them. The reactions could sometimes be rather unexpected.

Grandma, you look like a policeman (P4, interview 2, p. 12) and Someone asked me if I was planning to go flying and thought I was wearing a parachute (P3, interview 1, p. 9).

Some interviewees were not bothered that it was visible or that there were reactions and questions from those around them.

#### Personal relationship

When the interviewees had been told that they were going to receive a spinal orthosis that could strengthen their back muscles, a number of them stated that they expected it to make their backs straighter and reduce their back pain. Some interviewees hoped it would be a turning point in their lives and that using it to train the back muscles would become a daily routine. A number of them expected it to provide support for their backs and that it would be properly adjusted and feel comfortable. At the end of the trial, several interviewees stated that their expectations had been met. One interviewee stated that her expectations had been too high as she had hoped for a straighter back and relief from her back pain, which had not happened.

During the trial, the spinal orthosis was initially referred to as a corset. To establish what associations the interviewees had with the word corset, they were asked to describe their thoughts. It was associated with good posture and the hope of alleviating and improving back pain by providing strain relief but also with weakened muscles. They did not have positive associations with this word. It was something that was frightening, that pinched, chafed and had boning, buttons and push buttons, and something that was used in the olden days. One interviewee stated:

So you had to start with the boning that sits here and then it squeezes you in here and causes chafing here and chafing there (P4, interview 1, p. 4).

The interviewees’ perception changed and became more positive when they received information about how the spinal orthosis worked and how it was supposed to be used. Once they had used it for some time and experienced some form of effect (either positive or negative), a number of interviewees stated that they had developed a personal relationship with the spinal orthosis. Some described it as a long-term relationship or that it had become part of who they are, or it was like a jacket. It was described as something that was invaluable and wonderful and that they would miss it if it were no longer available.

I have positive feelings about the corset. It’s like a good friend that you can take out either before you need it or when you start to feel fatigue in your back. So you know it’s there and it feels very positive. (P2, interview 5, p. 8)

Over time, a negative relationship had developed with the orthosis and stopped using it because she thought it was complicated and did not add anything positive to her daily life.

I became so annoyed that it had to be adjusted every five steps so now it just hangs and takes place in my wardrobe. (P1, interview 5, p. 2

The interviewees were asked to describe their thoughts on how and when they would use the spinal orthosis going forward. The interviewees who were satisfied and felt it made daily activities easier had a positive attitude towards continuing to use it. One interviewee was unhappy with the fit but was willing to try again if it was refitted. A few interviewees had given up and stated that their back problems had not improved. They found the orthosis difficult to take on and off and did not intend to continue using it.

## Discussion

### Summary of the results

This qualitative interview study with focus groups provided a deeper understanding of what women with osteoporosis and back pain in primary care felt about using a spinal orthosis. Although there were similarities and differences between the interviewees’ experiences, the study showed that an individually adapted spinal orthosis could develop into *a long-lasting friendship that provided support and help in daily life*. But it needed to be adaptable and designed based on the individual’s needs. The three main categories, i.e. *impact on daily life*, *individual adaptation* and *personal relationship*, were clearly differentiated from each other but had an interdependency. All three categories were involved when the spinal orthosis was perceived as an aid that reduced an individual’s fatigue and back pain and facilitated daily life. These three categories also related to the interviewees who perceived the spinal orthosis to be cumbersome, obstructive and that it failed to provide symptom relief.

The spinal orthosis had a clear *impact on their daily lives*. Several interviewees stated that a spinal orthosis could make them feel like their back was stronger and that it could increase their level of physical activity. It could also provide some symptom relief and the perceived fatigue in their back could decrease. The spinal orthosis could also cause a perceived increase in support for the back and improved posture. Their postures could also be maintained, even after the spinal orthosis had been removed. This could be due to increased activity in the back muscles, which was described as muscle memory. These experiences find some support in previous quantitative studies in which an activating spinal orthosis was used that produced increased back extensor strength and reduced muscle pain after 6 months of treatment [10, 12, 13].

In our study, the spinal orthosis was described as being able to improve walking ability and extend walking distance. One explanation could be that the individual felt steadier and more confident and that it took longer for back fatigue to set in. This explanation is partially supported by a qualitative study showing that walking ability was improved for older individuals with thoracic kyphosis who used an activating spinal orthosis, as well as a study in which a spinal orthosis was used in rehabilitation after a vertebral fracture [25, 26]. The experiences of the interviewees were also supported by a study that showed that training of the back extensor muscles played an important role in relieving back pain, preventing the development of kyphosis and making it easier to perform daily activities for individuals with osteoporosis and back pain, with a vertebral fracture [2, 3]. The spinal orthosis was perceived by several interviewees as a training tool and that it could be a stimulant for training, which could conceivably have positive effects. When using a spinal orthosis results in increased physical activity, it may improve the well-being of an osteoporosis sufferer as physical activity can be perceived as being a tool for health [27]. The spinal orthosis has previously been used in the acute or subacute phase of rehabilitation for vertebral fractures as several studies have shown that back pain decreased and back strength improved [10, 11, 14–16]. We wanted to study what women seeking primary care with osteoporosis, fatigue pain and chronic back pain felt about using a spinal orthosis in their daily lives. In our study, it was found that women seeking primary care usually benefitted from an activating spinal orthosis as a treatment for their condition. In several quantitative studies involving the use of an activating spinal orthosis, the findings indicated increased back muscle strength, reduced back pain and reduced thoracic kyphosis, improved balance and walking ability, as well as improved health-related quality of life [13, 25, 26, 28, 29]. Increased back extensor strength can also prevent future vertebral fractures [6, 7]. In our study, these results are supported by the description the interviewees gave about using a spinal orthosis. Thus, this could serve as a supplement to other types of training the back extensor muscles in order to make everyday life easier for older women with osteoporosis and back pain.

The *individual adaptation* category showed the importance of regular adjustment of the orthosis. This study showed that the acclimation process and acceptance of a spinal orthosis could be facilitated through proper fitting and information on how it worked, as well as how and when it could be best used. An acclimation process can also be seen in other types of assistive devices, such as walking aids, in which studies show that support and information are needed in order to make it easier for the individual to accept a new assistive device that makes daily activities easier [30]. One problem with the spinal orthosis was that it tended to ride up during physical work, particularly work above shoulder height. Thus, when prescribing a spinal orthosis, it may be important to have regular follow-ups regarding how it is being used and what effect it is expected to have. There is also some support for the idea that follow-up of a prescribed assistive device may impact its continued use [31].

In the *personal relationship* category, after a period of use, a personal relationship with the spinal orthosis (either positive or negative) was developed. This could be interpreted as an acclimation process associated with experiencing benefit and help in daily life. When an assistive device is needed, the individual may perceive themselves as being no longer independent. In our study, it was considered important to be able to continue with normal everyday activities as independently as possible. This description is further supported in a study in which women with osteoporosis and back pain described a sense of independence as being important to their quality of life [32]. The process of accepting an assistive device may also involve an individual accepting that their body may change through osteoporosis, with reduced height and increased thoracic kyphosis. The knowledge that they have a fragile skeleton may cause them to perceive themselves as being weak, with a reduced work capability. This notion has been shown in a study in which patients looked at pictures of a fragile skeleton [33]. Our study indicated that if the spinal orthosis provided symptom relief and was comfortable to wear, then a personal relationship could develop in such a way that it became like “a friend” that provided support and a sense of security in everyday life when performing various activities. This personal relationship with the spinal orthosis could also be affected by positive or negative comments and reactions from those around the wearer as it could influence the decision regarding when and how to use the spinal orthosis going forward.

### Strengths and limitations

A strength of this study was that the group was relatively homogeneous. All women in the study had used the same product, a spinal orthosis, and the adherence to the use of a spinal orthosis among the interviewees was very good. They had initially been randomised to wear the spinal orthosis for 6 months in the RCT [13]. It is also a relatively limited area to discuss. Focus group interviews are a study design that fits well in a context, where several people have used the same product. Another strength was that the interviewees were held in a familiar location, which may have contributed to a safe and open discussion environment.

A limitation of this study could be that there was a risk that the interviewees skewed their description towards more positive experiences and did not emphasise negative experiences. All interviewees had met and been tested by the author at several occasions during the RCT. This may have led to a certain sense of loyalty and a desire to be accommodating in their answers. During the focus group interviews, neither the moderator nor the observer felt that this was a problem as many negative experiences emerged, especially in relation to the fit and self-adjustment of the spinal orthosis. Another limitation could be that there were only a few respondents in a couple of interviews. We assessed that there should not be any disadvantage because the subject was limited and few participants may also contribute with valuable information. Saturation was achieved with the number of respondents because the same experiences reappeared and nothing new was added.

## Conclusion

In older women with osteoporosis and back pain, an activating spinal orthosis could be perceived as being a “close friend” and a support in their everyday lives. It was important for the spinal orthosis to be well adjusted to the back and followed up regularly by the spinal orthosis prescriber and an orthopaedic technician. Support during the acclimation process was important, providing an opportunity to discuss when and how the spinal orthosis could be used.

## Electronic supplementary material

ESM 1 (DOCX 27 kb)

ESM 2 (DOCX 14 kb)
