# Supplementary material for: Experiences of using an activating spinal orthosis in women with osteoporosis and back pain in primary care
Source: Arch Osteoporos. 2020 Oct 29;15(1):171. doi: 10.1007/s11657-020-00754-z (PMC7595975; doi:10.1007/s11657-020-00754-z)
Supplement: Supplementary file 1 — (DOCX 27 kb) [file 11657_2020_754_MOESM1_ESM.docx]

Appendix 1. Examples of meaning units, condensed meaning units, codes, sub-categories, main categories and a theme from content analysis of using and managing an activating spinal orthosis.

| **Meaning unit** | **Condensed meaning unit** | **Code** | **Subcategory** | **Main category** | **Theme** |
| --- | --- | --- | --- | --- | --- |
| **Examples** | | |  |  |  |
| You suddenly remember how your posture was, in the orthosis and how you wanted to correct it. | Remember the posture want to recreate | Remember the posture | Better posture  Support  Stronger back  Pain relief  Training equipment  More reliable body | **Impact on daily life** | **“A well-adapted spinal orthosis could develop into a long-lasting friendship that provides support and help in daily life”** |
| I often wear it when I take the subway since I feel that it provides the support I need for my back. | Wearing it when riding the subway provides support for the back | Provides support for the back |  |  |  |
| Yes and I do think that the feeling of being tired in the back when I work too much at the kitchen counter is gone. Or rather, I put on the orthosis as soon as that feeling arrives and after that it becomes much better. | Orthosis helps against tiredness in the back. | Helps against tiredness in the back |  |  |  |
| The pain in my back I had before got so much better. | Back pain gets much better | Back pain gets better |  |  |  |
| I have never been able to any weight training before but thanks to the corset I’ve been able to start doing just that. | The corset has helped been able to start training muscle strength | Helps to been able start training muscle strength |  |  |  |
| I feel much safer, when wearing the corset, as it prevents me from making any straining movements that hurts my back. It feels like the corset becomes an insurance. | Feel safer in some way it prevents me making crazy movements. Becomes like an insurance. | The orthosis is a factor of safety |  |  |  |
| But it is little uncomfortable since it goes from down there to up here. | Little uncomfortable goes up and down | Uncomfortable goes up and down | Self-adjustment  Fitting | **Individual adaptation** |  |
| I needed several adjustments done since I’ve lost 7cm of my height. Because of that the corset came too far up the groin. | 7 cm came too far into the groin | Adjustment needed |  |  |  |
| I also think positively about the corset. I think of it as a close friend that you can use even if you don’t need to. | Positive feelings like a nice friend for the spinal orthosis | Positive feeling like a nice friend | Relationship over time  Expectations  Notions of the orthosis  Thoughts of the individual and reactions from those around them Thoughts about continuing the use of the orthosis | **Personal relationship** |  |
| The orthosis gave hope in some way, as it would relieve the pain and improve. | The orthosis gave hope. It could relieve and improve | Hope of relief and improvement |  |  |  |
| In my mind I saw my mother, who wore a corset, the one with the buttons and hooks. | In my mind a corset with buttons and hooks | Corset with buttons and hooks |  |  |  |
| There was one person who asked if I would fly away and thought I wore a parachute | Asked if flying away and if wearing a parachute | Asked – flying away – thought parachute |  |  |  |
| I should try to wear it when I'm out walking | Try to wear it when out walking | Wear it when walking |  |  |  |
