# Supplementary material for: Experiences of using an activating spinal orthosis in women with osteoporosis and back pain in primary care
Source: Arch Osteoporos. 2020 Oct 29;15(1):171. doi: 10.1007/s11657-020-00754-z (PMC7595975; doi:10.1007/s11657-020-00754-z)
Supplement: Supplementary file 2 — (DOCX 14 kb) [file 11657_2020_754_MOESM2_ESM.docx]

Appendix 1

**Interview guide**

Please describe your thoughts about the spinal orthosis?

Please describe how it feels to wear the spinal orthosis?

Please describe how the spinal orthosis has influenced your daily life?

Please describe your thoughts when you first heard the word corset?

Please describe your thoughts about the word corset today?

Please describe how it is to put on and take off the spinal orthosis?

Please describe the feeling in your back when you are wearing the spinal orthosis?

You have been wearing the spinal orthosis for six months - Please describe how it

feels in your back today?

Please describe your thoughts about the continued use of the spinal orthosis in future?
